# Disinfection of Hospital Sink Drains Enriches Pseudomonadota and Efflux Pump-Mediated Antibiotic Resistance in Reestablished Biofilms

**DOI:** 10.21203/rs.3.rs-7888495/v1

**Published:** 2025-10-27

**Authors:** Kate Bowie, Irvan Luhung, Taylor Burke, Scott Roberts, Richard Martinello, Mark Gerstein, Jordan Peccia, Hannah Healy

**Affiliations:** Yale University; Yale University; Yale University; Yale University; Yale University; Yale University; Yale University; Harvard T.H. Chan School of Public Health

## Abstract

Antimicrobial resistant pathogens and associated infections represent major public health threats affecting healthcare facilities, with sink drain biofilms serving as reservoirs for many of these bacteria. Despite attempts at sink drain biofilm disinfection and removal, drain biofilms inevitably regrow, and disinfection may shape the returning microbial communities and their resistance profiles. We applied culture-based and metagenomic approaches to study these drain disinfection effects on microbial community abundance, taxonomy, and antimicrobial resistance in operational hospital sinks. Drain biofilms regrew to baseline densities in approximately four days. Regrown biofilms contained more viable carbapenem-resistant bacteria and were dominated by Pseudomonadota, including *Cupriavidus* and *Pseudomonas*. Long-read sequencing revealed an increase in multidrug efflux pump genes after disinfection, which confer broad resistance to antibiotics and disinfectants. This work provides mechanistic insights into how disinfection influences sink drain biofilm ecology and the enrichment of antimicrobial resistance, with implications for infection prevention strategies in healthcare environments.

## Introduction

Multi-drug resistant hospital-acquired infections (HAIs) cost an estimated $1.9 billion in the United States annually,^1^ and treatment is increasingly more complex with rising antimicrobial resistance (AMR). Recent research has produced substantial evidence supporting sink drains as reservoirs of antimicrobial resistant bacteria (ARB), and drain ARB have been linked to AMR HAI outbreaks,^2–7^ with established pathways of dispersion from drains into hospital rooms during sink use.^8^ Sink drains promote the persistence of ARB through the growth of dense biofilm formation, which fosters close microbial interactions and facilitates horizontal gene transfer, a process by which bacteria can exchange antibiotic resistance genes.^9^ Environmental investigations into sink drain biofilms have identified ARB, such as multidrug-resistant *Pseudomonas aeruginosa*, *Cupriavidus pauculus*, *Acinetobacter baumannii*, and *Stenotrophomonas maltophilia*,^8^ many of which are listed as priority AMR pathogens by the World Health Organization (WHO) and United States Centers for Disease Control and Prevention (CDC).^10,11^ Given that healthcare sink drains play a role as reservoirs for priority ARB, interventions that prevent the growth of resistant pathogens in sink drains are critically needed. Equally important is understanding how such interventions alter the microbial ecology of drain biofilms, since biofilm dynamics and taxonomic composition directly influence the persistence and dissemination of antimicrobial resistance.

Drain disinfection is met with the challenge of ensuring sufficient contact time with vertical pipe walls. Recent foaming disinfectants have addressed that issue and have been gaining popularity in healthcare settings; however, guidance on application frequency and evidence of impacts in real-world settings is lacking. A recent study on one such product demonstrated efficacy against a variety of bacterial, yeast, and viral pathogens, and reduced overall bacterial abundance when applied to real sink drain biofilms every 4–5 days.^12^ Despite temporary success in reducing microbial abundance in drains with typical disinfectants,^13,14^ previous work has demonstrated that biofilms can reestablish relatively quickly and that disinfection in other contexts can preferentially select for antibiotic resistant organisms.^15,16^ These interventions are typically evaluated only in terms of bulk microbial load reduction, leaving their ecological and resistance-related impacts poorly understood. Addressing this gap is essential because interventions may not simply eradicate pathogens but instead, lead to regrowth that reshapes the microbial ecology and AMR of drain biofilms. Whether disinfection reduces clinically relevant resistance genes, selects for stress-tolerant organisms, or reshapes taxonomic diversity in sink drain biofilms remains largely unknown.

**Figure 1:**
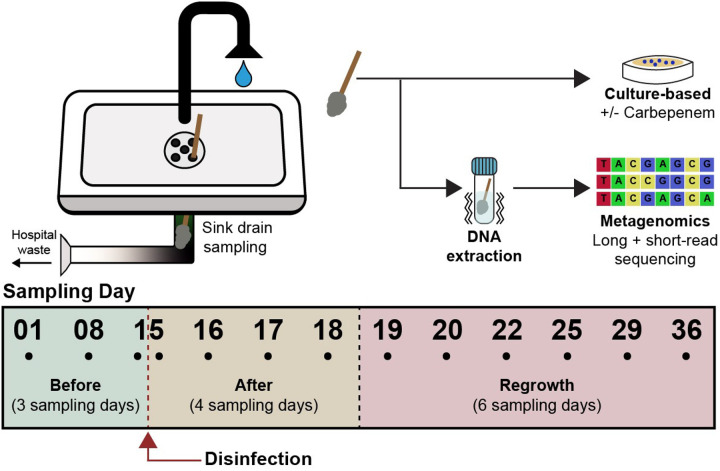
Overview of experimental design and sampling scheme across experimental phases.

Here, we used culture-based assays in combination with short- and long-read metagenomics to investigate how the microbial communities in hospital sink drain biofilms respond to a foaming disinfectant with active ingredients of hydrogen peroxide, octanoic acid, and peroxyacetic acid. We evaluated the sink drain biofilms of 19 operational sinks at Yale New Haven Hospital over 36 days. Biofilms were sampled prior to disinfection to identify baseline abundance and taxonomy, then sampled at several timepoints for three weeks after treatment (Fig. 1). We examined the viability and abundance of the microbial community through culture using agar with and without antibiotics to measure total and carbapenem-resistant bacteria (n = 156 samples). To better characterize the community members of the sink biofilms, we utilized short-read metagenomic sequencing (n = 119 samples). Lastly, long-read sequencing was applied to resolve the host context of antibiotic resistance genes (ARGs), providing insight into which bacteria carried resistance determinants and whether they were on chromosomes or mobile elements (n = 24 samples). This overall approach allowed us to link changes in resistance phenotypes to genomic mechanisms and taxonomic dynamics, providing new insight into how interventions may unintentionally favor reduced microbial diversity and broad-spectrum resistance in critical healthcare reservoirs.

## Results

### Disinfection reduced bacterial abundance followed by regrowth that enriched for carbapenem-resistant bacteria

**Figure 2:**
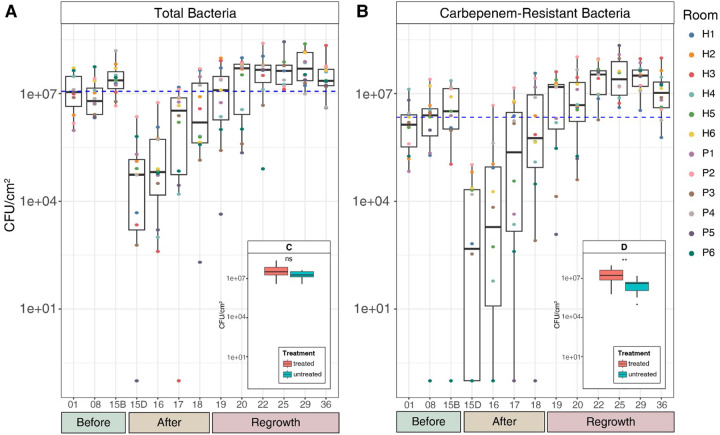
(A) Cultured total bacteria and (B) carbapenem-resistant bacteria from sink drain biofilms by study day, where drains were disinfected on Day 15. Sink rooms are designated with “P” for patient or “H” for hallway. Sampling days were separated into experimental phases: before (days 1–15B, B = before), after (days 15D-19, D = disinfection) and regrowth (days 20–36). Dashed blue lines represent the median CFU/cm^2^ of the first three timepoints (‘before’ phase) for each media type, and x-axis sampling days are not to scale. (C) Comparison of cultured viable bacteria from treated (red; n=12) and untreated (blue; n=7) sink drain biofilms cultured during the regrowth phase from total and (D) carbapenem-resistant bacteria (p < 0.01, Wilcoxon Rank Sum test).

We evaluated the sink drain biofilms of 19 unique sinks (12 treated, 7 untreated) on a hematology and oncology unit that typically cares for patients with hematopoietic stem cell transplants at Yale New Haven Hospital. Of the treated sinks, six sinks were located in patient rooms while the other six were located in hallways outside patient rooms, but still within the clinical unit. In the 156 samples collected, we assessed abundance of viable total bacteria and carbapenem-resistant bacteria by counting colony forming units (CFU) of homogenized samples spread onto ChromAgar Orientation plates (no carbapenem) and ChromAgar KPC (with carbapenem) plates, respectively. We designated three phases based on bacterial abundance and growth patterns surrounding treatment: baseline bacterial abundance before treatment (‘Before’), reduced bacterial abundance following treatment (‘After’), and regrowth period of bacterial abundance returning to at least baseline levels (‘Regrowth’, Fig. 1). At baseline (“before” disinfection), the median drainpipe surface loading of total bacteria was 1.2×10^7^± 9.2×10^6^ CFU/cm^2^, while the median of carbapenem-resistant bacteria was 2.2×10^6^± 6.7×10^6^ CFU/cm^2^. Both types of media reflected a two- to three-log reduction in colony-forming units immediately following disinfection (Fig. 2), in which many samples had no detectable colonies. Biofilms began to regrow after the single drain treatment, reaching the initial bacterial abundance after four days. Although bacterial abundance increased during the regrowth phase relative to baseline, it was not significantly different from that of the seven untreated control sink drains, suggesting that external factors contributed to the observed increases across all sinks (Fig. 1C).

Disinfection led to elevated carbapenem-resistant bacteria in the regrowth phase relative to baseline. The resistant fraction of bacteria was highest in the regrowth phase (median 50.0% IQR [19.2%, 81.8%], p = 0.05) and lowest before treatment (median 15.7% IQR [6.6%, 43.8%], after: median 26.1% IQR [5.8%, 61.5%]). The abundance of carbapenem-resistant bacteria in treated sinks was significantly higher than in the untreated sinks during the regrowth phase (Fig. 1D). These trends of initial reduction of bacterial abundance post-treatment followed by regrowth with enriched antimicrobial resistance were consistent across both hallway and patient-room sinks (**Supplementary Fig. 1**).

Treatment led to enrichment of Pseudomonas and Cupriavidus and decreased diversity in reestablished biofilms

**Figure 3:**
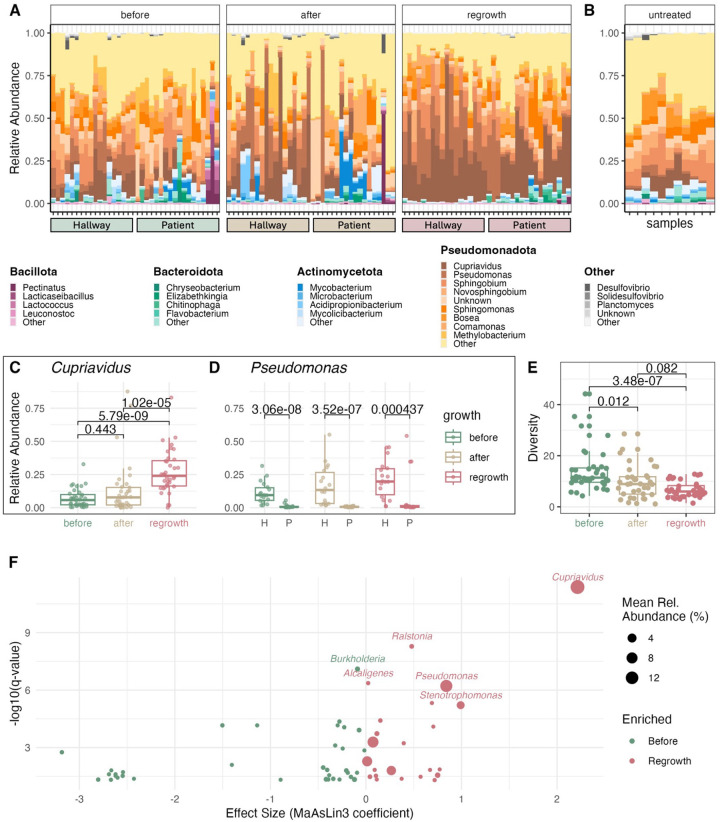
(A) Stacked relative abundance bar plot at the genus level of the sink biofilm sample microbial communities separated into experimental phases (before, after, and regrowth after disinfection). (B) Stacked bar plot of the untreated control sink drain biofilm communities with similarity to the samples before disinfection. (C) Boxplot of relative abundance of *Cupriavidus* across experimental phases. (D) Boxplot of relative abundance of *Pseudomonas*across the experimental phases, separated into patient (“P”) and hallway (“H”) sink drain biofilms. (E) Boxplot of the Inverse Simpson index across experimental phases with p values displayed above brackets. (F) Volcano plot comparing microbial abundance in the before vs. regrowth phases. The x-axis shows MaAsLin3 effect size, and the Y-axis shows significance (−log10 q-value). Each point represents a genus significantly associated with the transition from the baseline (before) to the regrowth phase. Genera with positive (red) effect sizes are more abundant in the regrowth phase, while those with negative (green) effect sizes are more abundant before treatment. Point size indicates mean relative abundance across samples, and the six genera with the lowest p-values were labeled.

To evaluate the impact of treatment and regrowth on microbial community taxonomy, we sequenced a total of 123 samples via Illumina short-read sequencing, which included samples from three collection days in each experimental phase, two negative controls, two positive controls, and seven untreated control sinks. The sink drain biofilms were taxonomically diverse, consisting of 41 assigned phyla and 1,648 assigned genera (Fig. 3A). The four most abundant phyla in the sink drain biofilms were Pseudomonadota, Actinomycetota, Bacteroidota, and Bacillota. The top five most abundant genera across all experimental phases were *Cupriavidus*, *Pseudomonas*, *Sphingobium*, *Novosphingobium*, and *Comamonas* (Fig. 3A, **Supplementary Fig. 2**), which are commonly detected in hospital water systems.^17–19^

The biofilm microbial community that regrew after disinfection was significantly different from the biofilm community before disinfection. *Cupriavidus* and *Pseudomonas* dominated in the regrowth phase, which coincided with a significant decrease of alpha diversity across metrics (Inverse Simpson index, p = 0.00003, Kruskal-Wallis, Fig. 3C–E; **Supplementary Table 1**). Additionally, we found a significant difference in the beta diversity of the microbial communities when comparing before disinfection to the regrowth phase (Bray-Curtis, p < 0.001, PERMANOVA, **Supplementary Fig. 3**), but it should be noted that there were significant differences in the reads generated from each experimental phase (p = 1.74 × 10^−5^, Kruskal-Wallis, **Supplementary Fig. 4**). Next, we investigated the specific taxa contributing to the overall composition changes by examining differentially abundant taxa throughout the three experimental phases (Fig. 3F, **Supplementary Table 2**). Our analysis revealed that *Cupriavidus, Ralstonia*, *Pseudomonas*, and *Stenotrophomonas* were significantly higher during regrowth, while *Burkholderia* was significantly lower (MaAsLin3, p < 0.05, Fig. 3F). *Moritella* was the genus with the lowest p-value (p = 3.42 × 10^−302^), although likely a statistical artifact and contaminant, as *Moritella* has not been documented to be in hospitals, pipes, nor near humans,^20^ and was removed from the analysis.

Genera containing frank and opportunistic pathogens were consistently detected in sink drain biofilm microbial communities, with higher overall abundance of pathogen-containing genera after treatment. Using CZID’s 2024 pathogen list,^21^ we found 73 unique genera in our sink drain biofilm communities (**Supplementary Table 3**). There was a median of 69 pathogen-containing genera (IQR: 67, 70 pathogens) detected per sink drain biofilm and no significant differences in number detected across the experimental phases. The top 5 pathogen-containing genera detected were *Pseudomonas*, *Stenotrophomonas*, *Mycobacterium*, *Enterobacter*, and *Mycolicibacterium*, all associated with water systems.^22^ The relative abundance of all pathogens was significantly higher directly after disinfection and in the regrowth phase compared to before treatment (p = 0.03, Kruskal-Wallis, **Supplementary Fig. 5**).

### Sink location shaped biofilm community composition and response to treatment

Sink location (in a patient room versus in a hallway) can influence its use, where mechanistic factors including temperature, relative humidity, and nutrient inputs can impact microbial community composition.^19,23^ We found changes in composition over the experimental phases were occasionally inconsistent between patient and hallway sinks (Fig. 3D), despite no significant differences in reads generated by sample type (i.e., hallway or patient room sinks, **Supplementary Fig. 11**). Although patient and hallway sinks shared the same top five phyla and same top genus, *Cupriavidus* (Fig. 3C), hallway sinks maintained significantly higher relative abundance of *Pseudomonas* over the sampling period (Fig. 3D, p = 1.6×10^−13^). In select patient sinks there were large blooms of *Pseudomonas* during regrowth; however, the majority of patient sinks contained *Pseudomonas* at low levels (< 5%), as shown by the distribution in Fig. 3D. The beta diversity between hallway and patient sink drain biofilms were significantly different at each experimental phase, indicating location impacted community composition, and that room type should be incorporated as a covariate into downstream analysis (Bray-Curtis, p = 0.001, **Supplementary Table 4**). These location-specific differences likely reflect variations in sink use, including both frequency and type of activity, which in turn may influence the reestablished biofilm.

### Metagenomic shotgun sequencing revealed decreased overall abundance of antibiotic resistance genes through treatment phases

**Figure 4:**
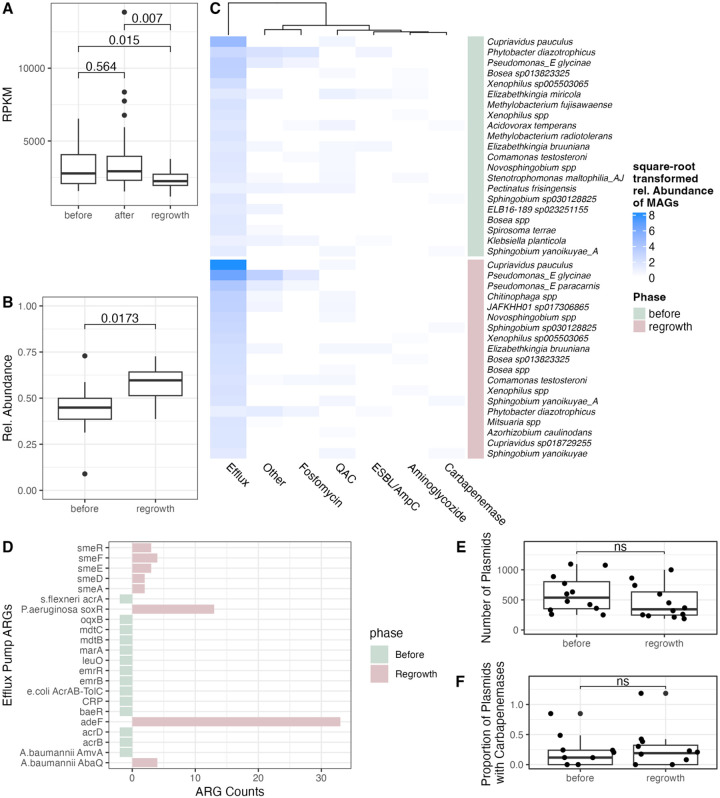
(A) Boxplot of the total RPKM of ARGs annotated in the shotgun metagenomic sequencing data by experimental phase, with p values displayed above brackets. (B) Boxplot of number of MAGs with ARGs before disinfection and in the regrowth phase. (C) Heatmap shows number of taxa with the ARG category of interest, described in **Supplementary Table 6**. MAGs were collapsed at the species level and were subset by those with >2 detections. Color intensity reflects the number of times the species-ARG pair was detected across samples. (D) Differential abundance (number of times ARG was found in a MAG) of ARGs associated with efflux pumps. Green represents ARGs that were more prevalent before disinfection while red represents ARGs more abundant in the regrowth phase after disinfection. (E) Boxplot demonstrating the number of plasmids before disinfection and in regrowth (ns = not significant where p = 0.16). (F) Boxplot demonstrating the proportion of plasmids with carbapenemases before disinfection and in regrowth (ns = not significant where p = 0.24).

In drain biofilm metagenomes, the five most abundant ARGs present were *adeF, qacG, qacJ, ANT(2)-la*, and *OXA-2*. These genes facilitate antibiotic resistance through different mechanisms, either by encoding efflux pumps with resistance to both antibiotics and disinfectants (adeF, qacG, qacJ),^24,25^ enabling aminoglycoside modifications (*ANT(2)-Ia*),^26^ or producing β-lactamases (*OXA-2*).^27^ The combined median reads per kilobase million (RPKM) of all ARGs in the sink drain biofilms was 2646.6 (IQR: 2036.0, 3312.5 RPKM). Although the culture results demonstrated higher phenotypic carbapenem resistance in reestablished biofilms, overall ARG abundance was significantly lower both after disinfection and during regrowth compared to before treatment (p = 0.005, Kruskal-Wallis, Fig. 4A). Despite the decrease in ARG abundance, there were no significant changes in alpha diversity of the ARGs throughout the experimental phases (**Supplementary Fig. 6B-C**). Carbapenem-resistance genes (median: 95.5 RPKM; IQR: 54.7, 197.2 RPKM) followed similar trends to overall ARG abundance, with lower abundance during the regrowth phase compared to both before and after disinfection (p = 0.001, Kruskal-Wallis, **Supplementary Fig. 7**).

### Disinfection enriched bacteria with chromosomal antibiotic resistance genes

Long-read sequencing enables the contextualization of antibiotic resistance genes in host bacteria, lessening the likelihood of detecting ARGs from free DNA fragments not associated with live or pathogenic bacteria.^28^ We sequenced biofilms from all treated sink drains using PacBio long-read sequencing from two collection dates: before disinfection (day 8, n = 12) and during regrowth (day 29, n = 12) and analyzed only complete metagenome assembled genomes (MAGs). We detected no significant differences in the number of MAGs recovered from samples before or after disinfection (mean 11.7 ± 4.8 vs 10.3 ± 1.9, respectively). Within these MAGs, we detected 96 unique ARGs belonging to 27 different drug classes and categorized into 6 distinct resistance mechanisms (**Supplementary Table 5**). We annotated an average of 3.5 ± 3.6 ARGs per MAG, including the 9.1% of MAGs without any ARGs. The top 5 most abundant ARGs by relative abundance were *adeF*, *vanT gene in vanG cluster*, *ArnT*, *acrB*, and *mdtB*, which differ from our short-read shotgun metagenomic results beyond the most abundant ARG (*adeF*).

The relative abundance of MAGs carrying ARGs increased significantly after disinfection (Fig. 4B), despite no significant change in the total number or ARGs (**Supplementary Fig. 8**, p > 0.05), indicating an enrichment of antibiotic-resistant bacteria (Fig. 4B). Of the MAGs containing ARGs, we found *Cupriavidus pauculus* and *Pseudomonas_E glycinae* to be the most abundant species, followed by *Phytobacter diazotrophicus* and *Elizabethkingia bruuniana*, all of which have been reported to cause nosocomial infectious except for *Pseudomonas_E glycinae* (**Supplementary Fig. 9**).^17,29–31^

We found carbapenemases to be rare and detected them in only four species during the regrowth phase (Fig. 4C, **Supplementary Table 6**). As previous work has demonstrated that carbapenemases are rarely chromosomal and are primarily located on plasmids,^33^ we extended our analysis from only MAGs to also include plasmids. We took all sequences outside of the MAGs and classified them as chromosomal or plasmid, then annotated with ARGs similar to our previous analysis. We found each sample had a median of 436.5 plasmids (IQR: 260.8, 749.3). Although not statistically significant, there was a decrease in the number of plasmids from before treatment to the regrowth phase, further supporting the hypothesis from short-read sequencing results (Fig. 4A) that disinfection may remove non-chromosomal DNA (p = 0.16, Fig. 4E). Narrowing our analysis to carbapenemases, we found no significant difference in the proportion of plasmids carrying carbapenemases before and after disinfection (p = 0.24, Fig. 4F). Together these observations highlight that mechanisms beyond plasmid-borne carbapenemases, or carbapenemases in general, likely underpin phenotypic resistance in hospital sink drain biofilms following disinfection.

### Multi-drug efflux pumps dominated antibiotic resistance in sink drain biofilms after disinfection

The majority of ARGs in the sink drain biofilms were associated with efflux pumps, which can confer low-level, broad-spectrum resistance. Overall, 83.7% of MAGs and 77.9% of species contained at least one ARG classified as an efflux pump. Both the percent of MAGs with efflux pumps and percent of species with efflux pumps increased significantly after treatment (p = 0.03, p = 0.008 respectively). This pattern aligns with the phenotypic increases in carbapenem-resistant bacteria observed in culture, despite the low abundance of canonical carbapenemases.^32^ Specifically, the efflux pump gene *adeF* was highly prevalent and detected across nearly all species, suggesting it may be a core resistance gene in these biofilms (Fig. 4D). The second-most abundant ARG was also an efflux pump, *Pseudomonas aeruginosa soxR*. We re-examined the shotgun metagenomic data to confirm whether this enrichment of efflux pumps was also apparent; however, no significant difference in efflux pump RPKM was observed across growth phases (**Supplementary Fig. 10**).

## Discussion

Effective infection prevention approaches are an essential component of patient care. Hospital sink drains frequently harbor biofilms, which serve as established reservoirs of pathogens and have been implicated in HAIs.^2–4^ The effects of sink drain disinfection as a means for controlling biofilm growth remains unclear, and evidence supporting its use is limited. In this study, we investigated how a disinfectant for drain biofilms influenced the microbial communities in 12 treated hospital sink drains. Through culturing, we found that disinfection effectively disrupted the biofilm communities and temporarily decreased microbial load (Fig. 2, “after”). Despite all sinks being the identical model, there was variability in disinfection efficacy, with viable cell count reductions ranging from 1–2 log, likely due to biofilm heterogeneity. Organic matter can accumulate over time, sometimes clogging drains, which would likely require several treatments or physical means to remove. After disinfection, bacterial communities regrew to baseline abundance in approximately four days. These findings parallel prior work demonstrating that repeated disinfection at 4–5 day intervals is required to maintain long-term reduction of biofilm biomass.^12^

The use of the disinfectant resulted in a strong selective pressure to the biofilm microbial communities that reduced diversity and enabled a few taxa to dominate. The metagenomic shotgun sequencing results revealed major compositional changes to the biofilm that consisted of decreased alpha diversity and enrichment of *Cupriavidus* and *Pseudomonas*, which are clinically relevant genera.^17,34,35^ A previous study involving the application of peracetic acid disinfectant to sink drain biofilms demonstrated that application resulted in a significant increase in *Pseudomonas spp*. during regrowth and a continued presence of antibiotic-resistant organisms in the drains, agreeing with our results.^36^ Long-read sequencing further resolved enriched taxa in our study, identifying *Cupriavidus* as *C. pauculus* while the dominant *Pseudomonas* showed 95.82% average nucleotide identity to *Pseudomonas_E glycinae*, a species previously documented only in soybean rhizospheres.^31^ This average nucleotide identity value suggests the recovered MAGs represent a distinct, sink-adapted species within the *P. glycinae* complex. Members of *Pseudomonas* produce extracellular polymeric substances (EPS) which promote biofilm formation and can inhibit fungal growth,^37^ features that may provide a competitive advantage within the sink drain environment. The concurrent dominance of *Cupriavidus* and *Pseudomonas* likely reflects both intrinsic tolerance to disinfectants and their ability to recolonize niches exposed by the disruption of extracellular matrix material.^34,38^ Moreover, *C. pauculus* has recently been recognized as an emerging opportunistic pathogen capable of causing infections in immunocompromised individuals.^17^

Importantly, resistant bacteria became proportionally more abundant as the biofilm regrew after disinfection, with higher overall abundance of resistant bacteria in treated sinks relative to untreated sinks. The significant increase in the fraction of viable carbapenem-resistant bacteria during regrowth underscores the selective pressure exerted by disinfection, raising the possibility that disinfectant use may favor resistant subpopulations.^15,16^ Our long-read sequencing demonstrated increases in the abundance of MAGs with ARGs, and those ARGs were primarily associated with multidrug efflux pumps (Fig. 4). Efflux pumps confer low-level multidrug resistance, often providing a survival advantage in biofilm settings where exposure to disinfectants, heavy metals, and other stressors selects for broad stress-tolerance mechanisms.^39^ Specifically, during regrowth we detected increases in the resistance gene *adeF*, which is part of the Resistance-Nodulation-Division family and encodes broad antibiotic resistance.^24,40^ *adeF* was found in a diverse array of taxa, including *Cupriavidus pauculus*, *Pseudomonas_E glycinae*, and *Elizabethkingia bruuniana* (Fig. 4C). All hits were classified as ‘strict’ by CARD, suggesting the presence of true orthologs. Additionally, our results suggest that treatment may have removed extracellular DNA carrying ARGs. We observed a decrease in total ARG abundance and a reduction in plasmids, even as chromosomally encoded ARGs in surviving MAGs increased. This shift implies that disinfectant pressure selectively eliminates mobile resistance elements while favoring taxa with intrinsic, chromosomally encoded resistance systems, further reinforcing the dominance of *Cupriavidus* and *Pseudomonas* after disinfection.

The observed increase in efflux pumps may explain the increase in phenotypic carbapenem resistance, despite no corresponding increase in the carbapenemase genes. Although the culture-based analysis demonstrated an increase in carbapenem-resistant bacteria, short-read shotgun metagenomics unexpectedly revealed an overall decrease in ARGs as well as a decrease in carbapenemase genes. Using our long-read metagenomic sequencing data, we examined both chromosomal ARGs in complete MAGs as well as ARGs on plasmids but found very few carbapenemases. The only carbapenemase we consistently detected was OXA-2, which has been debated in the literature and is not universally recognized as a true carbapenemase.^27,41^ These findings were similar to other studies in which carbapenemases were either not detected at all, or in the minority of their cultured bacteria or isolates.^32,42,43^ Instead of carbapenemases, we discovered that many MAGs had ARGs encoding multi-drug efflux pumps (Fig. 4C). A potential explanation is the bacteria likely did have phenotypic carbapenem resistance through the acquisition of multidrug efflux pumps rather than carbapenemases. This is consistent with experimental findings where disinfectant pressure promoted efflux pump overexpression,^44^ which can in turn result in carbapenem resistance.^45^

This research highlights how different methodological approaches can lead to different interpretations of antimicrobial resistance. Culture-based approaches are the norm in clinical settings, whereas sequencing and molecular methods are becoming increasingly common for environmental surveillance. Metagenomic approaches capture all DNA in a sample, including DNA from viable but not culturable bacteria, lysed cells, or extracellular fragments, thus they can capture more of the community but reflect genetic material not necessarily associated with viable bacteria. In this study, culture-based approaches enabled assessment of phenotypic resistance and viability, while sequencing results enabled identification of resistance mechanisms. Long-read metagenomic sequencing, in contrast with short-read metagenomic sequencing, enabled us to place ARGs in genomic context and distinguish those carried on bacterial chromosomes from those on plasmids, which is critical for understanding their potential for horizontal transfer and resistance mechanisms. However, simply counting ARGs can be misleading, since not all detected genes are expressed, functional, or relevant under clinical conditions, which has been demonstrated in a number of studies.^46,47^ Functional context, expression, and bacterial viability are necessary to link sequencing data to actual resistance phenotypes. Hence, investigators should be aware of tradeoffs, and the answer may be a combination of culture and sequencing-based approaches.

Taken together, hospitals need to balance the short-term need for pathogen removal in drains with long-term consequences of antimicrobial resistance propagation. These results demonstrate the enrichment of Pseudomonadota with broad efflux-mediated resistance has important implications for hospital infection prevention. Efflux pumps not only confer multidrug resistance but may also facilitate persistence under disinfectant pressure, enabling long-term colonization of sink drains. This combination of persistence and resistance maintains a reservoir from which opportunistic pathogens can disseminate to vulnerable patients. Thus, while disinfection remains a key tool for reducing biofilm biomass, its infrequent or sporadic application may inadvertently select for communities with heightened resistance potential. Although drain disinfection may be necessary in an outbreak scenario, we believe establishing the right frequency and duration of disinfection is critical for controlling sink drain biofilms and related pathogens, noting that inconsistent drain disinfection may come with a tradeoff of increased antimicrobial resistance.

The design of this study provided insights into immediate community shifts in the weeks following a single treatment. Future research is needed on dynamics during and after repeated applications over longer intervals. Additionally, larger-scale studies are needed to generalize findings. Finally, our threeweek follow-up may not have fully captured biofilm community stabilization, and it is unknown how long the elevated antimicrobial resistance and community shifts would have persisted. Despite these limitations, our results highlight key mechanisms by which disinfection can reshape sink biofilm communities and potentially increase antimicrobial resistance risk. This study lays the foundation for future work exploring alternatives to HAI strategies beyond disinfection. A variety of methods have been attempted for drain biofilm removal, including pipe heating/sonication, boiling water flushes, and even pipe removal and replacement, although these approaches are often costly, time-consuming, and short-term. Alternatives include exploring variations in sink design, such as utilizing different drainpipe materials, using germicidal ultraviolet light,^48^ and even introducing sinkless rooms,^49^ or testing probiotic-based interventions to outcompete pathogens in the sink drain.^50,51^ Ultimately, our findings underscore the need for infection control strategies that effectively manage sink biofilm dynamics, but also reduce conditions or pressures that drive the emergence and spread of antimicrobial resistance.

## Methods

### Sample collection

The study was conducted on a single floor (constructed 2009) focused on the care of patients with hematologic malignancies, particularly those with hematopoietic stem cell transplants, at Yale New Haven Hospital in New Haven, CT, USA. Twelve treated sinks and seven untreated sinks were included in the study. Treatment consisted of one application of disinfectant to sink drains, described below. Of the treated sinks, six sinks were in patient rooms (P1, P2, P3, P4, P5, P6), and six sinks were in the hallway directly outside patient rooms and in patient care areas (H1, H2, H3, H4, H5, H6). All sinks were handicap sinks of the same make and model (Chicago Faucets automatic model 116.517.AB.1). Treated sinks were sampled at three timepoints over two weeks prior to treatment and ten timepoints over three weeks after, while the seven untreated sinks were primarily sampled on the final two sampling days. As part of routine hospital protocol, all sinks were subjected to daily cleaning of outer surfaces including the counter and sink bowl with either a hydrogen peroxide or bleach-based disinfectant.

The disinfectant used in this study was Virasept (EcoLab), a product approved by the United States Environmental Protection Agency for drain disinfection in 2020. Active ingredients include 3.13% hydrogen peroxide, 0.099% octanoic acid, and 0.05% peroxyacetic acid. Virasept was applied as directed with the associated foamer, ensuring sufficient contact time of ingredients with vertical pipe biofilms. The foam was injected into the drain until foam began spilling over the drain cover, about 60 mL per sink. The disinfectant was left to incubate for five minutes, with additional foam applied if it started to drain. Sinks were not used during incubation and electronic faucets were prevented from activating. After five minutes, the sinks were flushed for at least 45 seconds, until there was no visible disinfectant present. Biofilm samples were collected using BD Liquid Amies Elution Swab (Eswab) Collection/Transport System. Sterile swabs were dipped in the Liquid Amies solution prior to sample collection. For drains, swabs were inserted approximately 7.6 cm and rotated across the pipe surface ten times (covering approx. 10 cm^2^). Swabs were then reinserted into the Liquid Amies solution, sealed, and transported to the lab on ice to be processed within 24 hours.

### Culture work

Swabs were vortexed in Liquid Amies solution to homogenize, then an aliquot of the solution was either plated or serially diluted in autoclaved tap water prior to plating. 50 μL aliquots of samples or dilutions were plated using the spread plate method with sterile spreader. Samples were cultured on KPC CHROMagar^™^ (“carbapenem-resistant bacteria”) and CHROMagar^™^ Orientation (“total bacteria”) plates. Plates (100 mm diameter) were made with 33 g/L chromagar media, and KPC supplement (400 mg/L) was added to the agar after cooling below 50°C. Sample dilutions were adjusted throughout the experiment (1x-100,000x) such that colonies were in a countable range. Colonies were counted after 24 hours of aerobic incubation at 37°C. Negative control plates spread with dilution water did not grow colonies.

### DNA extraction and clean-up

Swabs were extracted according to a protocol previously described.^19^ Briefly, swabs in Liquid Amies solution were bead-beaten for 15 minutes after adding lysis buffer and phenol-chloroform-isoamyl alcohol. The supernatant was removed after centrifugation and added into a 24-well plate with proteinase K for automated extraction with a Kingfisher Apex using the MagMax Microbiome TNA kit. After extraction, RNase A was added to a final concentration of 100 μg/mL and incubated at room temperature for 15 minutes prior to a column-based cleanup with the Genomic DNA Clean & Concentrator-10 kit (Zymo Research) to remove RNA.

### Short-read sequencing and bioinformatics

119 sink drain biofilm samples along with two negative swab controls and two mock community positive controls (Zymo Research) were sequenced at the Yale Center for Genomic Analysis (YCGA). YCGA completed the library preparation for 150 bp paired-end metagenomic shotgun sequencing at their facility then sequenced at a depth of 60 million reads per sample on the Illumina Novaseq 6000.

Illumina adapters were trimmed with cutadapt then data was prepared for processing using the Anvi’o Snakemake pipeline (version 8).^52,53^ Within Anvi’o, KrakenUniq was used to classify taxonomy using the KrakenUniq MicrobialDB database which contains sequences from the NCBI RefSeq database including bacteria, archaea, and viruses, and the human reference genome.^54^ Reads from each sample were individually assembled into contigs with a minimum length of 500 bp using Megahit.^55^ Prodigal was then used to convert the contigs from DNA to protein,^56^ then aligned to the CARD database with RGI to annotate ARGs.^57^ For functional analysis, the assembled contigs were first removed from Anvi’o then binned with metabat2 before importing back into Anvi’o.^58^ All data was exported into R for further analysis.

All samples generated sequencing data with a median of 47.6 million reads (range 28.2 to 113.1 million reads). Reads that could not be assigned at the Family level or higher were discarded. Taxa with a combined total of less than 1200 reads (0.005% of the median reads from all samples) were removed.^59,60^ To determine if a taxon was a contaminant, a ratio of the relative abundance in the negative controls to the relative abundance in biological samples was computed. If the ratio was greater than 10 in > 90% of samples, it was deemed a contaminant and removed from the dataset. Contaminants were visually inspected, and taxa that followed abundance patterns consistent with disinfection and regrowth were retained as true signal (e.g. Acidovorax). 16 taxa were labeled as a contaminant and were removed. After removal of reads unassigned to at least the family level and removal of contaminants, there was a median of 24.4 million reads per sample (range 11.4 million to 65.3 million reads, **Supplementary Fig. 11**). The number of reads generated from the negative controls were not significantly different from the biofilm samples (**Supplementary Fig. 12A**), however the composition of the controls did not match the true samples (**Supplementary Fig. 12B**). All subsequent analyses on the shotgun metagenomic data was done on taxa agglomerated at the Genus level.

To estimate the number of shotgun sequencing reads mapping to each ARG in each sample, we combined ARG annotations with contig-level coverage data. Specifically, contig coverage values were merged with ARG gene coordinates, and the length of each ARG was calculated from its start and stop positions. The number of reads per ARG was then estimated by multiplying the ARG length by the contig coverage and dividing by the average sequencing read length (150 bp). This approach provides an approximate read count for each ARG, facilitating downstream abundance analyses.

### Long-read sequencing and bioinformatics

24 samples were multiplexed and submitted for HiFi long-read sequencing using the PacBio Revio at the Keck Microarray Shared Resource at Yale University. Raw reads were assembled using metaFlye with the “meta” and “pacbio” flags.^61^ Samples were processed using PacBio’s HiFi MAG pipeline v2.0. Contigs greater than 500 kb with greater than 93% completion were considered final MAGs. The remaining long contigs were pooled with short contigs (< 500 kb) and underwent binning with both SemiBin2 and MetaBat2.^58,62^ The resulting bins were compared and merged using DasTool,^63^ then filtered with CheckM2 for > 70% completeness,^64^ <10% contamination, and < 20 contigs. All final complete MAGs were taxonomically classified using GTDB-Tk which generated final FASTA files and phylogenetic trees.^65^ For chromosomal antibiotic resistance gene annotation, Prodigal was used to predict the protein-coding genes from the final MAGs.^56^ Finally, RGI plus CARD was used in Strict mode to assign antibiotic resistance genes to the predicted proteins.^57^ ARGs with a > 75% percent identity and > 40% coverage were retained. We recovered an average of 11.0 ± 3.7 MAGs per sample.

The relative abundance of chromosomal MAGs was calculated as coverage multiplied by MAG length divided by the total number of bases in the sample. To determine ARG coverage, we used the length of the ARG and multiplied by the coverage of the MAG. ARG coverage was then divided by total number of bases in the sample to get ARG relative abundance. The ARG categories were first separated using the “Resistance Mechanism” output from CARD, and then further classified into specific resistances (i.e. fosfomycin) using prior work.^42^ To examine non-chromosomal ARGs, the unbinned contigs were annotated similarly to the complete MAGs with Prodigal followed by RGI plus CARD. Similar to prior environmental long-read sequencing work, we used PlasFlow to flag the unbinned contigs as either chromosome or plasmid.^66^ Final results were combined and loaded into R for data analysis.

### Data analysis

All data analysis was completed using R and Rstudio (version 4.5.0, 2025.05.0 + 496 respectively). Phyloseq (version 1.42.0) was used to analyze the short-read shotgun metagenomics data, including the negative controls and mock community positive controls, as well as the long-read sequencing data. MaAslin3 was used to evaluate differentially abundant taxa between after and regrowth compared to the before phase.^67^ The fixed effect was the experimental phase, while the random effects included both the specific sink and the sink location (i.e. hallway or patient) as they were not the main focus of this analysis.

All stacked barplots were generated using *microshades* (version 1.11).^68^ The vegan R package version 2.6.4 and *rstatix* version 0.7.2 were used for all statistical analyses. A Kruskal-Wallis test was used to test for significant differences between the experimental phases and relative abundance of specific taxonomy, followed by a pairwise Wilcoxon Rank Sum test with false discovery rate (FDR) correction. This study was deemed exempt from review by Yale University’s Institutional Review Board (Study #2000036758).

## Supplementary Material

Supplementary Files

This is a list of supplementary files associated with this preprint. Click to download.

- SupplementaryFigures.docx

## Data Availability

All custom codes used in the reported analyses are available on the GitHub page (https://github.com/katebowie/virasept). All software and package versions are detailed at the top of each code script. Sequencing data were deposited in NCBI SRA (PRJNA1310651).
